# Polyphenol-rich pomegranate fruit extract (POMx) suppresses PMACI-induced expression of pro-inflammatory cytokines by inhibiting the activation of MAP Kinases and NF-κB in human KU812 cells

**DOI:** 10.1186/1476-9255-6-1

**Published:** 2009-01-08

**Authors:** Zafar Rasheed, Nahid Akhtar, Arivarasu N Anbazhagan, Sangeetha Ramamurthy, Meenakshi Shukla, Tariq M Haqqi

**Affiliations:** 1 Department of Pathology, Microbiology, & Immunology, School of Medicine, University of South Carolina, 6439 Garners Ferry Road, Columbia, SC-29209, USA; 2 Department of Medicine, Division of Rheumatology, Case Western Reserve University, Cleveland, OH-44106, USA

## Abstract

**Background:**

Mast cells and basophils are multifunctional effector cells and contain plentiful secretary granules in their cytoplasm. These cell types are involved in several inflammatory and immune events and are known to produce an array of mediators including a broad spectrum of cytokines. Pomegranate fruit is rich in anthocyanins and hydrolysable tannins; a group of polyphenolic compounds shown to be potent antioxidant with anti-inflammatory activity. However, no studies have been undertaken to investigate whether a polyphenol-rich pomegranate fruit extract (POMx) inhibits the inflammatory activity of activated human mast cells and basophils. The aim of this study was to examine whether POMx modulates inflammatory reactions using human basophilic cell line KU812.

**Methods:**

KU812 cells were stimulated with phorbol-12-myristate 13-acetate plus calcium inophore A23187 (PMACI). The inhibitory effect of POMx on pro-inflammatory cytokine gene expression and production by stimulated KU812 cells was measured by quantitative RT-PCR, and cytokine-specific ELISA assays, respectively. Western blotting was used to analyze the effect of POMx on the activation of mitogen-activated protein kinases (MAPKs), and the nuclear factor (NF)-κB in PMACI stimulated KU812 cells. Effect on the activity of NF-κB was determined using Luciferase reporter assay. Significance of differences from control values were analyzed by means of standard statistical methods.

**Results:**

POMx significantly decreased PMACI stimulated inflammatory gene expression and production of interleukin (IL)-6 and IL-8 in KU812 cells. The inhibitory effect of POMx on the pro-inflammatory cytokines was MAPK subgroups c-jun N-terminal kinase (JNK)- and extracellular-regulated kinase (ERK) dependent. In addition, POMx suppressed the NF-κB activation induced by PMACI by inhibiting IκB-degradation in human basophil cells. POMx also suppressed the powerful induction of NF-κB promoter-mediated luciferase activity in transiently transfected KU812 cells.

**Conclusion:**

These novel pharmacological actions of POMx provide new suggestion that POMx or POMx-derived compounds may be of therapeutic use for the treatment of inflammatory diseases by suppressing mast cells/basophils activation.

## Background

Mast cells and basophils are known to play a central role in inflammatory and immune events [1]. Mast cells – derived mediators induce edema, destroy connective tissue, and are involved in lymphocyte chemotaxis and infiltration and in pathological fibrosis in rheumatoid arthritis (RA) joints. Moreover, these cells are involved in angiogenesis during inflammatory arthritis, and the proteolytic activity they produce is associated with cartilage destruction and bone remodeling [2]. The myeloid precursor cell line KU812 originally established from a patient with chronic myelogenous leukemia (CML) [3] has been shown in several published studies [4,5] to be a suitable model for studying the activation and degranulation of human mast cells [5-7]. Activation of these cells results in the degranulation accompanied by the production of chemical mediators such as histamine, proteases, metabolites of arachidonic acid and several inflammatory and chemotactic cytokines including interleukin (IL)-6 and IL-8. These molecules act on the vasculature, smooth muscle, connective tissue, and mucous glands, resulting in the recruitment of activated immune and inflammatory cells to the site of inflammatory lesion, thereby amplifying and sustaining the inflammatory condition [8]. The pro-inflammatory cytokines produced by the mast cells and basophils play an important role in the development of acute- and late-phase inflammatory reactions. Production of TNF-α, IL-6, IL-8, histamine and other inflammatory mediators by the activated mast cells could drive synovitis in RA [9,10], and it has been shown that mice deficient in mast cell activation were resistant to the induction of arthritis in the K/BXN model of rheumatoid arthritis [11]. These data suggest that inhibition of mast cell/basophil function could provide benefit in RA and other inflammatory diseases.

Mitogen-activated protein kinases (MAPKs) activated by various stimuli regulate the transcriptional activity of many genes involved in maintaining cellular homeostasis. Depending on the extracellular stimuli cell commences specific biological responses leading to differentiation, proliferation or apoptosis through the activation of MAPK signaling cascades. Activation of extracellular signal-regulated kinase (ERK), and *c-Jun *N-terminal kinase (JNK) by environmental stimuli play a significant role in the cytokine expression and appear to play an important regulatory role in inflammatory diseases including RA [12,13]. Nuclear factor (NF)-κB is a ubiquitously expressed transcription factor required for the expression of a number of inflammatory molecules [14] including TNF-α, IL-1β, and IL-6 [15]. For this reasons, NF-κB is an obvious target of emerging anti-inflammatory therapies [16].

Pomegranate (*Punica granatum *L, Punicaceae), is an edible fruit cultivated in many countries, including the United States and consumed around the world. It is well documented that the edible part of pomegranate is rich in anthocyanins and hydrolysable tannins, a group of polyphenolic compounds that possess antioxidant and anti-inflammatory activities [17]. Recently, pomegranate juice was found to revert the potent down regulation of the expression of endothelial nitric oxide synthase induced by oxidized low-density lipoprotein in human coronary endothelial cells [18]. Dietary supplementation of polyphenolic rich pomegranate extract to atherosclerotic mice was shown to inhibit significantly the development of atherosclerotic lesions [19]. It has also been shown that pomegranate extract can suppress NF-κB activation in vascular endothelial cells [20].

Recent studies from our laboratory have demonstrated that consumption of pomegranate may be of value in inhibiting inflammatory stimuli-induced cartilage breakdown and production of inflammatory mediators in arthritis and it may be a useful approach for the prevention of the onset and severity of inflammatory arthritis [21,22]. In the present study, we evaluated the potential of a standardized pomegranate fruit extract (POMx) as a therapeutic modality for inflammation using PMACI-stimulated human KU812 cells. Our results showed that POMx significantly inhibited the inflammatory stimuli-induced excessive production of IL-6 and IL-8 via modulation of the JNK- and ERK-MAPKs and NF-κB-dependent pathways.

## Methods

### Reagents and cell lines

Phorbol 12-myristate 13-acetate (PMA), calcium ionophore A23187 (Calcymycin; C29H37N3O6) were purchased from Sigma Chemical Co. (St. Louis, MO), and dissolved in DMSO. KU812 cells, RAW264.7 cells, RPMI-1640, DMEM were from American Type Culture Collection (ATCC) (Rockville, MD, USA). Antibodies were purchased from Cell Signaling Technology (Denver's, MA, USA) and Santa Cruz Biotechnology (Santa Cruz, CA, USA). Specific inhibitors for ERK (PD98059), and JNK (SP600125) were purchased from Biomol (Plymouth Meeting, PA). Inhibitor for NF-κB (MG-132) was purchased from Calbiochem (San Diego, CA, USA) Kits for performing cytokine specific enzyme-linked immunosorbent assays (ELISA) were purchased from R&D Systems (St. Paul, MN, USA). All other reagents/chemicals were of the highest analytical grade available.

### Chemical composition of POMx

POMx was produced from fresh pomegranate fruits (*Punica granatum *L., POM Wonderful brand) grown in California by Paramount Farms and was prepared by extraction of fruit residue after pressing for juice and solid-phase extraction to produce a powder with a high concentration of polyphenols. The powder extract used in this study contained on average 86.0% ellagitannins, 2.5% ash, 3.2% sugars, 1.9% organic acids as citric acid equivalents, 0.8% nitrogen, and 1.2% moisture. The approximate percent distribution of pomegranate polyphenols in POMx is as follows: 19% ellagitannins as punicalagins and punicalins, 4% free ellagic acid, and 77% oligomers composed of 2–10 repeating units of gallic acid, ellagic acid, and glucose in different combinations.

### Cell culture

KU812 cells were grown in RPMI-1640 medium supplemented with 10% heat-inactivated fetal bovine serum (FBS) and 1% penicillin-streptomycin at 37°C in 5% CO2. KU812 cells were pre-treated with POMx (20–100 μg/ml) for two hour prior to stimulation with 40 nM of PMA plus 1 μM of A23187 for different periods of time. POMx was dissolved in nuclease free double filtered distilled water, whereas PMA and A23187 were dissolved in DMSO.

### Real Time-PCR

Real time polymerase chain reaction (qRT-PCR) was used to quantify the expression of mRNA for IL-6 and IL-8 with expression of GAPDH as control. Total RNA was separated from KU812 cells by Quick Gene automated system according to the manufacturer's instruction (Quick Gene, USA). First-strand cDNA was synthesized using 1 μg total RNA and the SuperScript First Strand cDNA synthesis kit (Invitrogen, USA). Primers used were: IL-6 (F 5'-AAA TTC GGT ACA TCC TCG ACG GCA-3'; R 5'-AGT GCC TCT TTG CTG CTT TCA CAC-3'), and IL-8 (F 5'-AGA AAC CAC CGG AAG GAA CCA TCT-3'; R 5'-AGA GCT GCA GAA ATC AGG AAG GCT-3'); GAPDH (F 5'-GGA CTT CGA GCA AGA GAT-3'; R 5'-AGC ACT GTG TTG GCG TAC-3'). The amplification was performed using the qPCR core kit for SYBR Green (Qiagen, USA) and Step One Real Time system PCR (Applied Biosystems, Foster City, CA). Typical profile times used were initial step, 95°C for 15 min, followed by a second step at 94°C for 15 sec, 60°C for 30 sec and 72°C for 30 sec for 40 cycles with melting curve analysis. The level of target mRNA was normalized to the level of GAPDH and compared to control (untreated sample) and the values were calculated by 2^-ΔΔCT ^method, where ΔCT is the difference in threshold cycles for target and the housekeeping gene, and ΔΔCT is the differences in ΔCT and the threshold cycle for the control [23].

### Enzyme-linked immunosorbent assay (ELISA)

Cytokines produced in the culture medium were quantified by specific sandwich ELISAs. Briefly, KU812 cells were stimulated with PMA (40 nM) plus A23187 (1 μM) for 12 h with or without pre-treatment with POMx. The ELISA was performed using the culture supernatants according to the instructions of the manufacturer (R&D Systems). Plates were read at 450 nm using Synergy HT microplate reader (Biotek Instruction, Winooski, VT, USA).

### Western blot analysis

Stimulated and control KU812 cells were washed with cold PBS and lysed using the cell lysis buffer (50 mM Tris:HCl, pH 7.4; 150 mM NaCl; 1% Triton X-100; 0.1% SDS; 0.5% sodium deoxycholate; 1 mM EDTA; 1 mM EGTA; Complete^® ^protease and phosphatase inhibitors) as previously described [24]. Cytoplasmic and nuclear fractions were prepared as previously described [25]. Total lysate or nuclear/cytoplasmic fraction protein (45 μg/lane) was resolved by SDS-PAGE (10% resolving gel with 4% stacking) and transferred to nitrocellulose membranes (Bio-Rad). Membranes were blocked with blocking buffer containing non-fat dry milk powder in Tris buffered saline containing 0.1% Tween-20 (TBS-T), and probed with 1: 1000 diluted primary antibodies (Cell Signaling Technologies, Santa Cruz Biotechnology) specific for the target protein. Immunoreactive proteins were visualized by using 1:5000 diluted HRP-linked secondary antibodies and enhanced chemiluminescence (GE Healthcare, Milwaukee, WI, USA) [26]. Images were captured by using AFP-Imaging System (Minimedical Series, Elms Ford, NY, USA).

### Transient transfection and luciferase activity assay

Transient transfection and luciferase activity assay were performed as previously described [21]. Briefly, KU812 cells were seeded at 3 × 10^6 ^cells ml-1 in 12-well plate 4 h before the transfection in serum-free medium. Lipofectamin 2000 reagent (Invirtogen, Carlsbad, CA) containing the NF-κB luciferase reporter gene constructs, pNF-κB-LUC (Panomics) was added to the cell cultures according to the instructions of the manufacturer (Invitrogen, Carlsbad, CA). After 6 h of incubation, medium was replaced with fresh medium containing 10% FBS and antibiotics. Transfected KU812 cells were stimulated with PMACI for 20 h. In some experiments POMx (20–100 μg/ml) was added to the cultures for 2 h prior to PMACI-stimulation. Cells were harvested after 20 h stimulation and washed in ice-cold PBS before lysis in 100 μl of cell lysis buffer and the luciferase activity was determined according to the manufacturer's protocol (Luciferase Assay kit; Promega, Madison, WI), using a Lumat LB 9507 [Luminometer] Berthold Technologies, Germany). Luciferase activity was expressed as relative light units (RLU) per milligram of cell lysate protein.

### Statistical analysis

All statistical analyses were performed using Origin 6.1 software package (one paired two tailed *t*-test with one way ANOVA) and *P *< 0.05 was considered significant. Values shown are mean ± SD unless stated otherwise.

## Results

### Effect of POMx on cell viability and pro-inflammatory cytokines expression in activated KU812 cells

**Figure 1:**
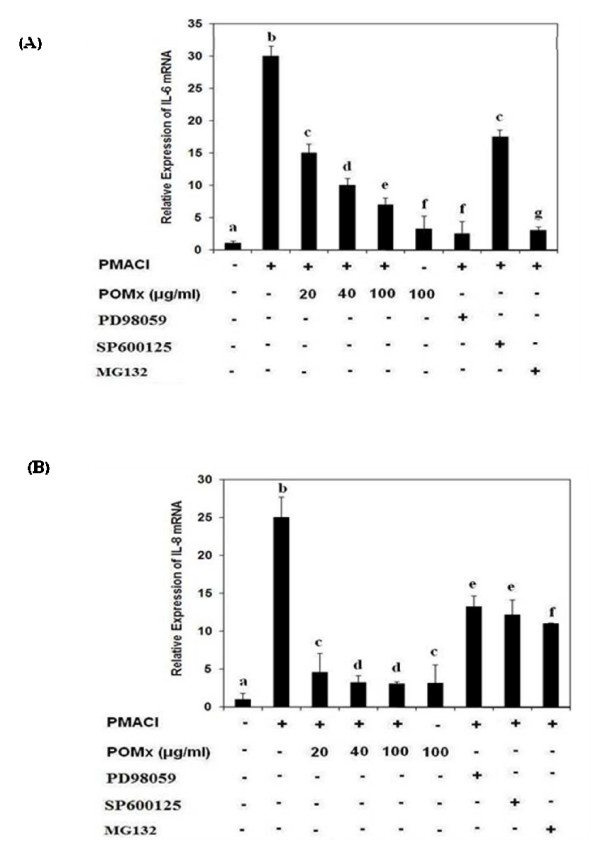
**Effect of POMx and specific inhibitors for MAPKs and NF-κB on the gene expression of pro-inflammatory cytokines in PMACI-stimulated KU812 cells**. KU812 cells (3 × 10^6 ^cells ml^-1^) were pretreated with POMx (20–100 μg/ml) for 1 h and stimulated by PMA (40 nM) plus A23187 (1 μM) for 4 h. The mRNA expression level of IL-6 (A) and IL-8 (B) was determined by quantitative reverse transcriptase PCR. The concentration of specific inhibitors of ERK (PD98059), JNK (SP600125) and NF-κB (MG-132) used in these studies was 50 μM, 10 μM and 100 μM, respectively. Values shown are Mean ± SD of four independent experiments and differ without a common letter *P *< 0.01.

**Figure 2:**
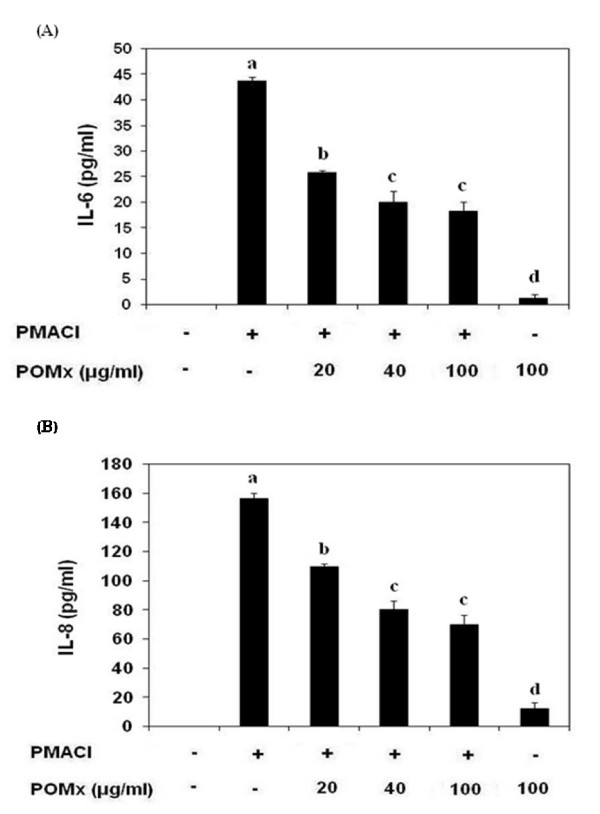
**Effect of POMx on the production of pro-inflammatory cytokines in PMACI-stimulated KU812 cells**. KU812 cells (3 × 10^6 ^cells ml^-1^) were pretreated with POMx (20–100 μg/ml) for 1 h and stimulated by PMA (40 nM) plus A23187 (1 μM) for 12 h. The production level of IL-6 (A) and IL-8 (B) was determined by a sandwich ELISA. Values shown are Mean ± SD of four independent experiments and differ without a common letter *P *< 0.05, *P *< 0.001.

We first examined the cytotoxicity of POMx on KU812 cells using Trypan Blue assay. POMx did not show cytotoxic effect up to a concentration of 300 μg/ml (data not shown). Next, we examined whether POMx could modulate gene expression of pro-inflammatory cytokines IL-6, and IL-8 induced by PMACI in KU812 cells. For these studies cells were pretreated with POMx (20–100 μg/ml) for 1 h, then stimulated with PMA (40 nM) plus A23187 (1 μM) for 4 h. As shown in Fig. 1A, and Fig. 1B, pretreatment with POMx dose dependently inhibited PMACI-induced gene expression of IL-6 and IL-8 as determined by quantitative RT-PCR. To confirm the effect of POMx on the production of pro-inflammatory cytokines, culture supernatants were assayed for cytokine levels using cytokine-specific ELISA. As shown in Fig. 2A and 2B, pre-treatment with 20–100 μg/ml of POMx significantly decreased the PMACI-induced IL-6 and IL-8 production in the culture supernatant of activated KU812 cells.

### Effect of POMx on the activation of MAPK

**Figure 3:**
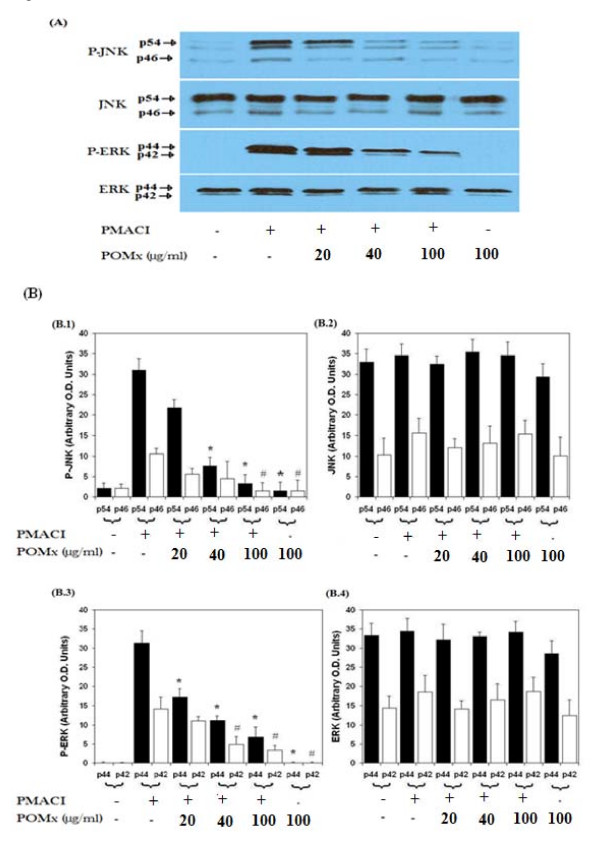
**Effect of POMx on the MAPKs phosphorylation in PMACI-stimulated KU812 cells**. After pretreatment with POMx (20–100 μg/ml) for 1 h at 37°C, KU812 cells (3 × 10^6 ^cells ml^-1^) were stimulated by PMA (40 nM) plus A23187 (1 μM) for 2 h, then the phosphorylation of JNK (p54/p46), and ERK (p44/42) was determined by Western blot analysis. Image were digitally captured and the band intensities (pixels/band) were obtained using the Un-Scan-It software and are expressed in arbitrary O.D. units. Data shown is cumulative of two experiments and the O.D. values are mean ± SD. *P < 0.001 Vs PMACI alone (P-JNK/ERK-p54), ^#^*P *< 0.001 Vs PMACI alone (P-JNK/ERK-p46).

Activation of MAPKs is intimately associated with the expression of pro-inflammatory cytokines. To determine whether the inhibition of IL-6 and IL-8 expression was mediated by inhibition of MAPK, we examined the effect of POMx on the activation of MAPKs in KU812 cells. KU812 cells were pretreated with POMx (20–100 μg/ml) for 1 h and then stimulated with PMACI for 2 h and cell lysate was analyzed by Western immunoblotting. Pre-treatment of KU812 cells with POMx attenuated the PMACI-induced phosphorylation of JNKp54/p46- and ERKp44/p42 (Fig. 3). No effect on p38-MAPK was observed (data not shown).

To further strengthen the relation of JNK- and ERK-inhibition by POMx and proinflammatory cytokines expressions in KU812 cells, we investigated the effects of pharmacological agents that inhibit JNK- and ERK. Treatment of KU812 cells with the selective JNK inhibitor, SP600125 (50 μM), and ERK inhibitor, PD98059 (10 μM), blocked the PMA plus A23187-induced IL-6 and IL-8 gene expression as determined by quantitative RT-PCR (Fig. 1A &1B). These data support the contention that inhibition of IL-6 and IL-8 expression by POMx in KU812 cells (Fig. 1A &1B) was mediated, at least in part, by the inhibition of PMACI-induced activation of JNK and ERK pathways.

### Effect of POMx on NF-κB activation

**Figure 4:**
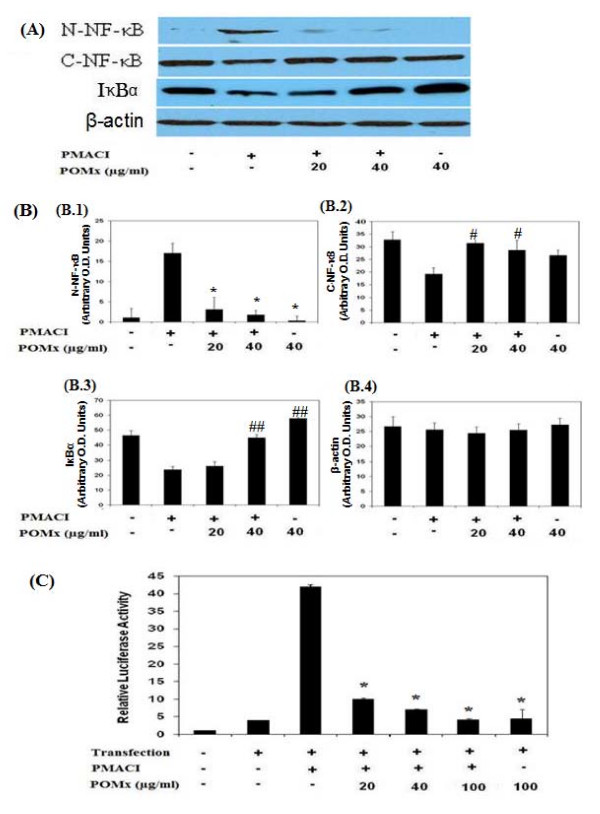
**Treatment with POMx inhibited the activation and DNA binding activity of NF-κB in PMACI-stimulated KU812 cells**. KU812 cells (3 × 10^6 ^cells ml^-1^) were pretreated with POMx (20, 40 μg/ml) for 1 h and stimulated by PMA (40 nM) plus A23187 (1 μM) for 2 h. (A) IκBα degradation and NF-κB translocation was analyzed by Western immunoblotting using antibodies specific for the p65 subunit of NF-κB (C-NF-κB, cytoplasmic NF-κB; N-NF-κB, nuclear NF-κB). (B) Band intensities were obtained as described above Data shown is cumulative of two experiments and the O.D. values (pixels/band) are mean ± SD. *P < 0.001 Vs PMACI alone (N-NF-κB), ^#^*P *< 0.05 Vs PMACI alone (C-NF-κB), ^##^*P *< 0.05 Vs PMACI alone (IκBα). (C) NF-κB luciferase assay: Cells were transiently transfected with the NF-κB luciferase reporter construct or empty vector and the NF-κB-dependent transcriptional activity was determined by luciferase activity using a commercially available kit (Promega). Each bar represents the mean ± SD of three independent experiments. *P < 0.001 Vs PMACI alone.

NF-κB is an important transcriptional regulator of inflammatory cytokines gene expression and plays a crucial role in immune and inflammatory responses. After the ubiquitination and phosphorylation of IκBα, the inhibitor is degraded and the NF-κB is translocated to the nucleus where it binds and activate the promoter of target genes. To further investigate the mechanism responsible for the inhibitory effect of POMx on pro-inflammatory cytokine expression such as IL-6, and IL-8, we examined the effect of POMx on NF-κB activation and translocation to the nucleus using Western blotting. Stimulation of KU812 cells with PMA plus A23187 induced the degradation of IκBα and nuclear translocation of p65 NF-κB (Fig 4A &4B). Pretreatment with POMx (20–100 μg/ml) inhibited the PMA plus A23187-induced degradation of IκBα and nuclear translocation of p65 NF-κB (Fig 4A &1B) in KU812 cells.

To determine whether POMx also inhibit DNA binding activity of NF-κB, we used a NF-κB-dependent gene reporter assay. KU812 cells were transiently transfected with a NF-κB-luciferase reporter construct or the empty vector. Exposure of cells to PMA plus A23187 enhanced the luciferase activity several fold in the cells transfected with the NF-κB-luciferase reporter construct. Increasing doses of POMx (20–100 μg/ml) significantly reduced the PMA plus A23187-induced luciferase activity (Fig. 4C). To further strengthen the relation of NF-κB pathway and the expression of IL-6 and IL-8 in KU812 cells, we next investigated the effect of a pharmacological agent, MG-132, a known inhibitor of NF-κB, on the expression of IL-6 and IL-8. Treatment of cells with the proteasome inhibitor MG-132 (100 μM), blocked the PMACI-induced IL-6 and IL-8 expression as determined by quantitative RT-PCR (Fig. 1A &1B). Together these results suggest that POMx exert its inhibitory effect on IL-6 and IL-8 expression via modulation of the activation and DNA binding activity of NF-κB.

## Discussion

Mast cells are emerging key players in the erosive and inflammatory events leading to joint destruction in inflammatory arthritis. Accumulation of mast cells in rheumatoid synovial tissue and their activation and degradation associated with pro-inflammatory cytokines and matrix degrading enzymes at cartilage erosion sites suggest that they could be usefully selected as a therapeutic target [2]. Mast cells are known to play a central role in inflammatory diseases as these cells contain potent inflammatory mediators, including histamine, heparin, proteinases, leukotrienes, and multifunctional cytokines, and their potential contributions to processes of inflammation and matrix degradation have become evident [27,28]. In response to diverse stimuli, human basophilic KU812 cells release an array of inflammatory cytokines and chemokines especially IL-6 and IL-8 which have the potential to cause inflammation and tissue remodeling [28]. IL-6 is a multifunctional cytokine that plays a key role in immune response, growth and differentiation of B- and T-cells, hematopoiesis and the induction of hepatic acute phase plasma proteins [29]. Increased tissue levels of IL-6 in diseases like arthritis [30], psoriasis [31], scleroderma [32], and delayed pressure urticaria have been demonstrated [33]. Binding of IL-6 to its receptor is known to induce IL-6-dependent signal transduction in mast cells and basophils [34]. IL-6 is also an important co-factor in IL-4 dependent IgE synthesis. IL-6 is also produced by mast cells and basophils and its local accumulation in arthritic joints is associated with the chronic response [35]. IL-8 is best known for its potent chemoattractant activity on neutrophils and T-cells and their functions [36]. Like IL-6, IL-8 has been shown to be increased in various inflammatory diseases, including arthritis, that are characterized by elevated mast cells number [35]. Interestingly, many reports have shown that human mast cells and basophils secrete both IL-6 and IL-8 [37,38].

Polyphenols are plant molecules entering in our bodies through diet. The relationship between polyphenol-rich food consumption and reduced possibility of being affected by some diseases has attracted increasing interest from consumers, food manufacturers and nutritional scientists. Fruit and vegetable consumption may prevent cancers [39] and stroke [40], whereas wine consumption may have similar effect in preventing coronary heart disease [41], and prostate cancer [42]. Soy consumption may have protective effects against cancerous cells [43] and osteoporosis [44] and tea polyphenols may prevent different cancers [45] and arthritis [46]. Pomegranate fruit is a rich source of polyphenols. Hydrolysable tannins are predominant polyphenols found in pomegranate juice and account for 92% of its antioxidant activity [47]. Pomegranate seeds are rich in sugars, polyunsaturated fatty acids, vitamins, polysaccharides, polyphenols, and minerals and have high antioxidant activity. When crushed and dried, the pomegranate seeds produce an oil with 80% punicic acid, the 18-carbon fatty acid, along with the isoflavone genistein, the phytoestrogen coumestrol, and the sex steroid estrone. The seed coat of the fruit contains delphinidin-3-glucoside, delphinidin-3,5-diglucoside, cyanidin-3-glucoside, cyanidin-3,5-diglucoside, pelargonidin-3-glucoside, and pelargonidin-3,5-diglucoside with delphinidin-3,5-diglucoside being the major anthocyanin in pomegranate juice [48]. Pomegranate fruit extract are also rich in oligomers which upon hydrolysis produce ellagic acid, which is a potent antioxidant, anticancer and antiatherosclerotic agent [22,49,50]. Studies have also shown that the antioxidant capacity of pomegranate juice is three times that of the popular antioxidant-containing beverages such as red wine and green tea, presumably due to the presence of hydrolyzable tannins in the rind, along with anthocyanins and ellagic acid derivatives [47]. In a comparative analysis, anthocyanins from pomegranate fruit were also shown to possess higher antioxidant activity than vitamin-E (α-tocopherol), ascorbic acid and β-carotene [51]. Pomegranate extract has also been shown to protect from NSAID and ethanol-induced gastric ulceration [52]. Repeated administration of high doses of a hydroalcoholic extract of pomegranate whole fruit or its constituent ellagitannin punicalagin were non toxic in the dosages commonly employed in traditional medicine systems [53,54]. Flavonoid rich fractions of pomegranate fruit extract have also been shown to exert antiperoxidative effect as their administration significantly decreased the concentrations of malondialdehyde, hydroperoxides and enhanced the activities of catalase, superoxide dismutase, glutathione peroxidase and glutathione reductase in the liver [55,56]. We recently reported *in vivo *efficacy of pomegranate constituents and/or their metabolites that become bioavailable after oral ingestion pomegranate fruit extract [22]. Here we show that POMx, a hydrolysable tannin rich extract of pomegranate, inhibited the gene expression and production of pro-inflammatory cytokines IL-6 and IL-8 by a human mast cell – like KU812 cells. The MAPK cascade is one of the important signaling pathways in an inflammatory response [57]. The signaling pathways characterized by MAPKs p38, ERK, and JNK, are known to play a potential role in the regulation of inflammatory response [58]. They are the key players in the molecular and cellular events associated with the pathogenesis of inflammatory arthritis and are being studied as a rational target of drug design for arthritis therapy [59]. In the present study, POMx specifically inhibited the PMACI-induced activation of JNKp54/p46- and ERKp44/p42-sub-groups of MAPK and inhibited the production of IL-6 and IL-8 by KU812 cells. In addition, JNK- and ERK-specific inhibitors, SP600125 and PD98059 also reduced IL-6 and IL-8 gene expression, respectively, in KU812 cells. These data suggest that compounds present in POMx have the potential to inhibit the inflammatory stimuli-induced JNK- and ERK-MAPK activation and inhibit the downstream IL-6 and IL-8 gene and protein expression.

Activation of the master transcription factor NF-κB leads to the coordinated expression of many genes that encode cytokines, chemokines, enzymes, and adhesion molecules involved in mediator synthesis and the further amplification and perpetuation of the inflammatory reaction [16]. Expression of IL-6 and IL-8 gene is dependent on the activation of transcription factor NF-κB [60]. Because suppression of NF-κB activation has been linked with anti-inflammatary activity, we postulated that POMx mediates its inhibitory effects on IL-6 and IL-8 expressions at least in part, through the suppression of NF-κB activity. Activation of NF-κB requires phosphorylation and proteolytic degradation of the inhibitory protein IκBα, an endogenous inhibitor that binds to NF-κB in the cytoplasm and its degradation expose the nuclear localization signal (NLS) and allows the NF-κB to translocate to the nucleus and bind the promoter of target genes [61]. In PMA plus A23187-stimulated KU812 cells, POMx inhibited the degradation of IκBα and nuclear translocation of the p65 NF-κB (Fig. 4A &4B). In addition, DNA binding activity of NF-κB as demonstrated by the reporter assays (Fig. 4C) was also inhibited in these cells. These data indicate that POMx attenuates the inflammatory stimuli-induced activation and DNA binding activity of NF-κB in KU812 cells. As IL-6 and IL-8 genes are NF-κB dependent genes, this also inhibit their expression and production in PMACI-stimulated KU812 cells.

Cytokines produced by mast cell and basophils are associated with the progress of inflammation. Both basophils and mast cells play a major role in the pathogenesis of inflammatory diseases by releasing several pro-inflammatory mediators. Our results suggest that POMx may regulate the pro-inflammatory cytokine expression and production by mast cells through different mechanisms. In view of the increasing prevalence of allergic and inflammatory diseases such as arthritis, asthma, allergic rhinitis, and eczema worldwide [16,28], there is a need for novel and safe treatment and or prevention option for the underlying inflammation caused by activation of mast cells and basophils [62]. Mast cells play different roles in the inflammation by the release of various chemokines and cytokines via different intracellular signal transduction pathways [62]. The results obtained in this study provide new evidence that POMx may contribute to the prevention and/or treatment of inflammatory diseases by inhibiting the activation of mast cells.

There is evidence from several studies that supplementation with POMx improves inflammatory symptoms *in vivo *and *in vitro *[21,48-52,56]. However, the molecular pharmacological basis for the observed effects has not been fully uncovered yet. Direct inhibitory effects of plant extracts or components in other systems have been reported [63,64], but few have addressed the question of bioavailability and activity of bioavailable constituents. In this regard results reported by Schaffer, et al [65] are important as they showed that after oral ingestion of pycnogenol human plasma contained bioactive compounds that inhibited the activity of COX-1 and COX-2 in an in vitro assay. We also wish to point out that the in vivo efficacy of the extract used here has already been shown by us in an animal model of inflammatory arthritis [21] indicating that after oral consumption pomegranate metabolites can exert anti-inflammatory effect in vivo. This gets strength from our studies showing that after oral consumption of a pomegranate extract, its constituents/metabolites become bioavailable and inhibit COX-2 activity, PGE_2 _and NO production in chondrocytes [22]. It is well documented that fruit or plant extracts are a complex mixture of various constituents and in most of the instances it is still not clear whether a single compound or a mixture of compounds is responsible for the reported effects [65]. However, evidence is accumulating that often related compounds present in a fruit or herb extract augment each other's biological effect. For example, it has been reported that ellagic acid and quercetin (both are also present in pomegranate) together exert a more pronounced inhibitory effect against cancer cell growth than either compound alone [66].

## Conclusion

Our is the first report that shows POMx inhibits the inflammatory activity of activated human mast cells like KU812 cells. The results of the present study indicate that POMx inhibits PMACI-induced pro-inflammatory cytokines production *via *inhibiting the gene expression. This is achieved by blocking JNK- and ERK-MAPK activation and NF-κB activation in human KU812 cells. POMx or POMx-derived compounds may be of value for the treatment of inflammatory diseases in which mast cells play an active role.

## Competing interests

ZR, NA, ANA, SR, MS declare that they have no competing interests. TMH has consulted for POM Wonderful.

## Authors' contributions

ZR, NA, ANA, SR, MS carried out the experimental work, collection and interpreted the data. TMH conceived of the study, its design, coordination, data interpretation and drafting the manuscript.
